# The importance of promoting scientific advocacy & outreach for trainees

**DOI:** 10.1038/s41386-023-01530-6

**Published:** 2023-01-11

**Authors:** Siara Kate Rouzer, Leanna Marie Kalinowski, Erin Taniyo Kaseda

**Affiliations:** 1 Department of Neuroscience & Experimental Therapeutics, Texas A&M School of Medicine, Bryan, TX 77807 USA; 2 Department of Community Health and Health Behavior, University at Buffalo, Buffalo, NY 14260 USA; 3 Department of Psychology, Rosalind Franklin University of Medicine and Science, North Chicago, IL 60064 USA

**Keywords:** Outcomes research, Translational research

Many scientific organizations, including the Society for Neuroscience, National Science Foundation, Nature Publishing Group, and American Association for the Advancement of Science, actively encourage their members to engage in scientific advocacy, describing public outreach as “a scientific imperative” and “key for the future of scientific research.” However, the authors of this article—three trainees specializing in neuroscience and neuropsychology - recognize that other early-career scientists may feel trepidation engaging in activities outside the laboratory or clinic. Outreach and advocacy are often undervalued as professional activities, and thus not rewarded or incentivized under traditional academic structures. Even with interest in outreach, early-career researchers may worry about time-commitments, whether activities will foster their career goals, and if their level of knowledge is adequate to promote science in a meaningful way. To address these uncertainties, we devised this article to explicitly state the benefits of scientific outreach, and to share a curated list of relevant outreach opportunities for trainees.

## Scientific outreach is advantageous to establishing a career in science

Across research disciplines, graduate students and postdoctoral fellows are expressly encouraged to pursue a career as a well-rounded scientist. Whether transitioning into research or non-research positions in academia, industries, government, or non-profit organizations, management and communication skills are most frequently utilized, as opposed to technical laboratory skills [1]. Engaging in scientific outreach provides opportunities to develop these translatable skills, facilitating adaptive communication, critical thinking, networking, and leadership. Community outreach and political advocacy, for instance, challenge trainees to speak about their work to audiences without their specialized knowledge. These same communication skills can be particularly useful for establishing collaborations with scientists outside of one’s field of expertise, and for closing the gap between basic science and clinical implementation. Outreach also encourages trainees to think of their work on a “grander scale” – i.e., implications of their data on health outcomes and scientific progress, a component of most research funding applications.

Encouraging trainees to share their research through press releases (e.g., *Newswise*, *New York Times*) can make neuroscience more accessible to the public *and* increases the likelihood their studies will be cited by other researchers [2, 3]. Even writing a tweet about a research publication increases that article’s attention/Altmetric score and citation numbers [4]. Finally, engagement in outreach provides opportunities for meeting scientists outside of one’s immediate network, which facilitates career advancement [5].

## Public engagement improves the perception of research and promotes public trust of scientists

Although academic research may often be perceived as detached from the public interest, there is an important reciprocal relationship between scientists, policy makers, and the public. In the past year, the U.S. federal government appropriated $165 billion for research and development [6]. Scientific funding is not the only critical interplay between science and government; policy can and does dictate areas of prioritization and deprioritization, influence of stakeholders, and communication of findings. Despite the heavy influence of policy on science, the majority of federal lawmakers come from backgrounds in law or business [7]. Thus, legislators are reliant on advocates and lobbyists to direct their attention and resources, with defense contractors, oil companies, banks, insurance companies, and churches making up many of the powerful and well-funded U.S. lobbies [8]. Communication between scientists and lawmakers can facilitate a stronger understanding of scientific priorities and big-picture issues, in addition to challenging myths, misunderstandings, and mistrust in science.

Recently, the politicization of major areas of scientific inquiry, including climate change and public health, have impacted the relationship between scientists, politicians, and the public. Engaging in outreach and advocacy as scientists has the power to address this widening gap. Among members of the public, scientifically literate citizens are better able to manage the criticisms, nuance, and uncertainty inherent in active scientific progress [9]. There is a responsibility to disseminate federally-funded research to taxpayers, as was reflected in the Biden administration’s recent policy to make taxpayer-funded work freely available to the public immediately upon publication [10]. While increasing access to science is important, interpreting and translating scientific results is a critical task for scientists to foster public trust.

## Advocacy and outreach facilitate trainee confidence and feelings of belonging

The public believes that scientists should engage in advocacy and increase their current levels of engagement [11]. Science is among the most trusted professions in society, and scientists’ continued engagement with the public is important for maintaining that trust. Importantly, this trust extends to pre-doctoral trainees, who often experience feelings of inadequacy and imposter syndrome. In reality, trainees are budding experts in their field; they often have master’s degrees, years of research experience, scientific publications, and are seen as trustworthy sources of information by policymakers and the public, particularly when they communicate firsthand experience with ongoing research. Further, as they are also constituents, trainees have power to influence change in their districts through communication with their elected officials.

Strategies for improving confidence in one’s ability as a scientist, and feelings of belonging, are crucial for recruiting and maintaining a diverse scientific workforce. For women and members of historically-excluded racial and ethnic groups, lack of confidence and sense of belonging are drivers for leaving academic research [12]. Engaging in scientific outreach and advocacy is one way that these factors can be diminished because these activities provide trainees with a sense of community and the feedback that their work and expertise are valued by society [13].

## Advocacy opportunities are diverse – there are ways for all to participate productively

**Fig. 1:**
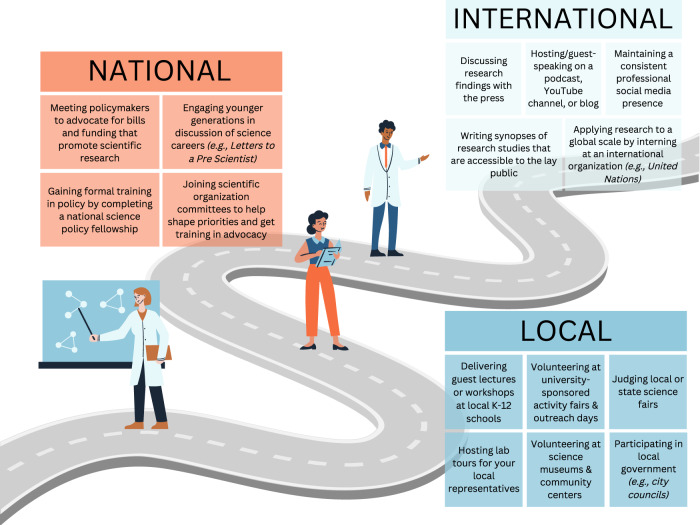
Examples of diverse outreach & advocacy opportunities for trainees. A summary figure of trainee opportunities for scientific engagement at the local, national and international level.

Despite the many benefits of performing advocacy and outreach, only 40% of graduate students and 17% of postdoctoral fellows report engaging in these activities on a regular basis. The most highly cited barriers to participation include lack of time, funding, and awareness of the opportunities that fit within their time and budgetary constraints [14]. Many trainees are unaware of the many fantastic options for neuroscience and neuropsychology trainees to participate in outreach - from local community engagement, to advocacy on a national level (Fig. 1*)*. Several of these opportunities require minimal time commitment—with some requiring as little as one day per semester – and are often already funded by universities and external organizations. Furthermore, there is much room for creativity and initiative, including trainees starting their own outreach programs [15].

To aid trainees in finding relevant outreach activities, the authors of this article have created a repository of outreach and advocacy resources, which will be updated regularly. As researchers who have spent years of their training engaging in advocacy, we hope to make it easier for trainees to establish themselves as scientist-advocates.
